# Assessing the protection elicited by virus-like particles expressing the RSV pre-fusion F and tandem repeated G proteins against RSV rA2 line19F infection in mice

**DOI:** 10.1186/s12931-023-02641-w

**Published:** 2024-01-04

**Authors:** Min-Ju Kim, Ki Back Chu, Su-Hwa Lee, Jie Mao, Gi-Deok Eom, Keon-Woong Yoon, Eun-Kyung Moon, Fu-Shi Quan

**Affiliations:** 1 Department of Biomedical Science, Graduate School, Kyung Hee University, Seoul, 02447 Republic of Korea; 2 Medical Research Center for Bioreaction to Reactive Oxygen Species and Biomedical Science Institute, Core Research Institute (CRI), Kyung Hee University, Seoul, 02447 Republic of Korea; 3 Department of Medical Zoology, School of Medicine, Kyung Hee University, Seoul, 02447 Republic of Korea

**Keywords:** Respiratory syncytial virus (RSV), rA2 line19F, Virus-like particles (VLPs), Pre-fusion F antigen, Tandem repeated G protein, Vaccine

## Abstract

Excessive pulmonary inflammation is the hallmark of respiratory syncytial virus (RSV) infection hindering efficacious RSV vaccine development. Yet, the vast majority of the experimental RSV vaccine studies use laboratory-adapted RSV strains that do not reflect the highly pathogenic and inflammatory nature of the virus found in clinical settings. Here, we re-evaluated the protective efficacy of the virus-like particle (VLP) vaccine co-expressing the pre-fusion (pre-F) protein and G protein with tandem repeats (Gt) reported in our previous study against the recombinant RSV rA2-line19F strain, which inflicts severe mucus production and inflammation in mice. VLP vaccine immunization elicited virus-specific serum antibody responses that mediated RSV rA2-line19F virus neutralization. VLP vaccine immunization promoted Th1 immune response development in the spleens and CD8 + T cell influx into the lungs of mice, which are essential for efficient viral clearance and dampened inflammatory response. When compared to the VLPs expressing only the pre-F antigen, those co-expressing both pre-F and Gt antigens conferred better protection in mice against rA2-line19F challenge infection. Overall, our data suggest that the pre-clinical VLP vaccine co-expressing RSV pre-F and Gt antigens can effectively protect mice against RSV strains that resemble pathogenic clinical isolates.

**Supplementary Information:**

The online version contains supplementary material available at 10.1186/s12931-023-02641-w.

## Introduction

Respiratory syncytial virus (RSV) is an infectious virus causing bronchiolitis and pneumonia in infants under the age of 2 throughout the entire world. The Centers for Disease Control and Prevention (CDC) has announced that generally 40,000 infants under 5 years of age are infected with RSV in the United States [1, 2]. To date, numerous RSV vaccine studies have undergone clinical trials with varying results. For example, Novavax’s nanoparticle-based maternal RSV vaccine failed to meet the phase 3 clinical trial endpoints [3, 4] while Moderna’s mRNA vaccine expressing the pre-fusion (pre-F) antigen demonstrated 83.7% efficacy in the elderly [5]. Recently, after decades of global effort, a pre-F subunit vaccine manufactured by GlaxoSmithKline was approved for clinical use [6].

The vast majority of the RSV vaccine studies reported to date revolved around using laboratory-adapted A2 or the Long strains for challenge infection, which poorly reflect the currently circulating RSV strains. In support of this notion, RSV cultured in primary human bronchial epithelial cell cultures produced large G proteins around 170 kDa, which significantly differs from the 95 kDa protein produced in most immortalized mammalian cell lines such as HEp-2. This feature was reported to have important implications for differential RSV infectivity in cell lines [7]. Differences in cytopathogenicity, inflammatory response induction, and growth kinetics between clinical virus isolates and laboratory-adapted RSV A2 strains were also reported. Specifically, clinical isolates A/2001/2–20 and A/2001/3–12 exhibited greater disease severity, airway mucin expression, and lung viral loads compared to RSV A2 strain in BALB/c mice [8]. This is also the case for the Line 19 strain, a clinical isolate acquired from an infant at the University of Michigan [9], which incurs severe RSV infection symptoms characterized by airway hyperreactivity, excessive inflammation, and mucus production [10–12]. There are other strain-specific differences in Line 19 which differs from the A2 or the Long RSV strains. While A2 tends to induce IL-10 cytokines, the Line 19 strains generally induce IL-13 cytokines. Furthermore, the Line 19 strain also substantially enhances *Gob5* and *Muc5ac* gene expressions that result in goblet cell hyperplasia *in* vivo, which are not observed upon RSV A2 infection [8, 11]. Therefore, studies utilizing either the highly pathogenic clinical isolates or a virus strain inducing similar disease severity must be conducted to evaluate the true protective efficacy of RSV vaccines as this approach would exemplify how the vaccines would fare in the clinical settings.

One earlier study revealed that substituting the fusion (F) protein of RSV A2 with the F antigen of RSV line 19 strain (rA2-line19F) resulted in substantial mucin expression and airway hyperresponsiveness in BALB/c mice, which were not observed from the laboratory-adapted RSV A2 or the Long strains [13]. Further work elucidated the role of several unique amino acid residues observed in line 19 F protein including enhanced fusogenic activity and mucin production, which resembles those observed in several clinical isolates [11, 13, 14]. Despite their importance, only a handful of studies attempted to investigate the efficacy of vaccines expressing the pre-fusogenic (pre-F) conformation of the RSV F antigens against the RSV line 19 strain [15, 16]. Moreover, none of the studies currently reported to date attempted to evaluate the protection elicited by the highly immunogenic virus-like particle (VLP)-based vaccines against RSV line 19. In our previous study, we assessed the protective efficacy of VLPs co-expressing the pre-F protein and the G protein tandem repeats (Gt) against RSV A2 strain in mice. We found that Gt VLPs alone were capable of conferring protection against the RSV A2, but when pre-F antigen was co-expressed along with the Gt, substantially enhanced protection was observed in immunized mice following RSV A2 challenge [17, 18]. Although these VLPs were efficacious and provided adequate protection in BALB/c mice, the A2 strain used in the aforementioned study does not incur excessive inflammatory response or mucin production. To address this limitation of our previous works, we evaluated the immune response and the overall protection elicited by these VLP vaccines against rA2-line19F infection in mice. Our findings revealed that the VLP vaccines expressing both pre-F and Gt (pre-F + Gt VLPs) protected mice against clinically relevant rA2-line19F virus infection, thus suggesting its potential for further development.

## Materials and methods

### Animals and ethics statement

Six-week-old female BALB/c mice were purchased from NARA Biotech (Seoul, Republic of Korea). All experimental procedures involving animals were performed following the Kyung Hee University IACUC guidelines (permit ID: KHSASP-21-340). Immunization and blood collection were performed under mild anesthesia, which was induced and maintained with ketamine hydrochloride and xylazine. All efforts were made to minimize the number of animals used in the experiment as well as their suffering.

### Cells and virus preparations

To generate recombinant baculovirus (rBVs) and VLPs, *Spodoptera frugiperda* insect cells (Sf9) were maintained and used using a serum-free SF900-II medium (Invitrogen, Carlsbad, CA, USA). HEp-2 cells (ATCC, Manassas, VA, USA) were cultured using DMEM (Welgene, Daegu, Republic of Korea) supplemented with 10% heat-inactivated fetal bovine serum and 1% penicillin/streptomycin and maintained in a 37℃ incubator with 5% CO_2_. RSV rA2 line19F viruses were kindly provided by Dr. Moore and propagated using HEp-2 cells [13]. At 90–95% confluence, cells were infected with the RSV rA2 line19F strain at 0.1 MOI in serum-free DMEM for 2 days at 37℃, 5% CO_2_. Infected HEp-2 cells were harvested using a scraper, and centrifuged at 3,000 rpm for 15 min at 4℃. Cell pellets were resuspended in a small volume of serum-free DMEM and subsequently sonicated. After centrifuging the sonicated cells at 2,000 rpm for 10 min, 4℃, the supernatant fraction containing the viruses was carefully collected and titrated via plaque assay. Formalin-inactivated RSV (FI-RSV) and live RSV A2 virus immunization groups were included as controls and these viruses were prepared as described previously [18]. All virus samples used in this study were stored at -80℃ until use.

### Generation and characterization of VLPs

Recombinant baculovirus (rBV) constructs were acquired following the manufacturer’s instructions as outlined in the Bac-to-Bac Baculovirus Expression System (Thermo Fisher Scientific, Waltham, MA, USA). VLPs were assembled by transfecting Sf9 cells with the rBVs as previously described [18]. Prefusion-stabilized RSV A2 F construct described by Patel et al. [19] was used in this study, which contains mutations in the furin cleavage site II and the deletion of 10 amino acids (F137-V146) from the fusion protein. The codon-optimized pre-F construct which was cloned into the pFastBac vector was purchased from GenScript (Piscataway, NJ, USA). Gt antigen was custom synthesized by connecting the tandem repeat regions of the RSV G protein with 4 glycine linkers as described in our previous study [18]. In brief, codon-optimized genes were cloned into pFastBac vectors and subsequently transformed into DH10Bac competent cells. Bacmid DNA was acquired from successful clones and these were transfected into the Sf9 cells. After 3–4 days of incubation at 27℃, cell culture media containing the rBVs were carefully collected. Using this method, a total of three different recombinant baculoviruses (rBVs) were initially prepared in Sf9 cells for VLP assembly, each expressing the influenza M1, RSV pre-F antigen, or the RSV G protein with tandem repeat (Gt). The influenza M1 acted as a core protein for all of the assembled VLPs described in this study. VLPs were assembled by co-infecting Sf9 cells with the rBVs expressing the influenza M1 along with either pre-F or pre-F with Gt antigens (pre-F + Gt). RSV VLPs were characterized through western blots and transmission electron microscopy (TEM). The expression of VLP protein components was confirmed by western blots. Primary antibodies were diluted in Tris-buffered saline with 0.1% Tween-20. Pre-F, Gt, and influenza M1 proteins were detected using RSV fusion protein monoclonal antibody (Merck Millipore, MA, USA; 1:5,000 dilution), RSV positive sera from mice (1:600 dilution), and influenza M1 monoclonal antibody (Abcam, Cambridge, UK; 1:5,000 dilution; clone GA2B). For RSV-positive serum acquisition, mice were intranasally infected twice with 4 × 10^6^ pfu of RSV A2 strain at 4-week intervals. Horseradish peroxidase (HRP)-conjugated secondary anti-mouse IgG antibody was used as a secondary antibody and bands were developed using enhanced chemiluminescence. All images were acquired using ChemiDoc (Bio-Rad, Hercules, CA, USA). VLP morphologies were observed under TEM. After adsorbing VLPs and staining with 2% uranyl acetate on copper grids, images were acquired using Bio-High Voltage EM System (JEM-1400 Plus at 120 kV and JEM-1000BEF at 1,000 kV, JEOL Ltd., Tokyo, Japan).

### Immunization and challenge

Mice (n = 10 per group) were lightly anesthetized with isofluorane and intranasally immunized twice with 80 µg of pre-F or pre-F + Gt VLPs at 4-week intervals. For FI-RSV and live RSV control groups, mice were immunized once with either 50 µg of FI-RSV or 1 × 10^5^ pfu of RSV A2 virus, respectively. Four weeks after the final immunization, mice were challenge-infected with 1.2 × 10^5^ pfu RSV rA2 line19F strain through the intranasal route. At 5 days after challenge infection, mice were sacrificed for spleen and lung tissue sample collection. Lung tissues were homogenized and centrifuged. The supernatant fractions were collected to assess lung virus titer, while the pelleted cells were isolated into single cells via Percoll density gradient for flow cytometry use. Splenocytes were also homogenized using frosted slide glass. After centrifugation, supernatants were used to assess cytokine concentrations while splenocytes were used for flow cytometry. All supernatant fractions were stored at -80℃ until use.

### Antibody response detection in sera and lung

Four weeks after each immunization, sera were collected via retro-orbital plexus puncture to evaluate RSV-specific antibody responses and virus-neutralizing antibody titers as previously described [17, 18]. Virus-specific IgG and IgA antibody responses were assessed by enzyme-linked immunosorbent assay (ELISA). After coating 96-well plates with 200 ng/ml of FI-RSV dissolved in carbonate coating buffer overnight at 4℃, wells were blocked with 0.2% gelatin prepared in phosphate buffered saline with 0.1% Tween-20 (PBST). After incubating the plate with diluted sera (1:100 in PBS) or lung supernatants (1:10 in PBS) for 1.5 h at 37℃, HRP-conjugated anti-mouse IgG and IgA secondary antibodies (1:2,000 dilution in PBS; Southern Biotech, Birmingham, AL, USA) were inoculated into respective wells. Plates were incubated at 37℃ for 1.5 h. O-phenylenediamine substrate dissolved in 0.05 M citrate buffer with H_2_O_2_ was added to each well for color development and OD_490_ was measured using an EZ Read 400 microplate reader (Biochrom Ltd., Cambridge, UK).

### Detecting virus-neutralizing activity in sera

For virus neutralization assay, sera acquired from mice 4 weeks after boost immunization was used. Sera were inactivated by heating at 56℃ for 30 min and diluted 1:10, 1:50, 1:250, 1:500, and 1:1000 in PBS. Equal volumes of diluted sera and RSV rA2-line19F were mixed and incubated at 37℃ for 1 h. After incubation, mixtures were inoculated into a confluent monolayer of HEp-2 cells cultured in 24-well plates and subsequently incubated at 37℃ for 1 h. The mixtures were aspirated and cells were overlaid with 1% noble agar, then incubated for 3 days at 37℃. Plaque reductions from each well were compared with those of the control.

### Antibody secreting cell response (ASC) in splenocytes

At 5 days post-challenge infection, mice were sacrificed for splenocyte collection as previously described [20]. Isolated splenocytes were seeded in 96-well plates coated with RSV F and G protein mixture (2 µg/ml) and cultured in complete RPMI media for 5 days at 37℃. After discarding the supernatants, plates were incubated with HRP-conjugated anti-mouse IgG, IgA, IgG1 and IgG2a secondary antibody (1:2,000 dilution in PBS) for 1.5 h at 37℃. After adding the O-phenylenediamine substrate solution, OD_490_ was measured (EZ Read 400 microplate reader; Biochrom Ltd., Cambridge, UK).

### Flow cytometric assessment of pulmonary immune cell populations

Lung tissues were homogenized without enzymatic digestions involving dispase or collagenase and single cells were isolated via Percoll density gradient as described previously [20]. Flow cytometry was performed to determine the T cell and eosinophil populations in lung. Lung cells were stimulated with RSV F and G protein mixture purchased from Sino Biological (Beijing, China) at concentration of 2 µg/ml for 5 h at at 37℃. Afterward, Fc receptors were blocked with Fc Block™ (BD Biosciences, CA, USA; clone 2.4G2) and cells were stained with CD3e (FITC, clone 145-2C11), CD4 (PE-Cy7, clone RM4-5), CD8a (PE, clone 53 − 6.7), CD11b (APC, clone M1/70), Siglec F (PE, clone E50-2440), Ly6G (PE-Cy7, clone IA8), and CD125 (Alexa488, clone T21) antibodies (BD Biosciences, CA, USA). CD3, CD4, and CD8 surface markers were used to determine T cells, while the remaining 4 surface antibodies were used to identify eosinophil populations. Stained cells were acquired using a BD Accuri C6 Flow Cytometer (BD Biosciences, CA, USA).

### Cytokine production in the spleen

The inflammatory cytokine production was performed as previously described [21]. Isolated splenocytes were counted and grown with RSV F and G protein mixture (2 ug/ml) for 5 days at 37℃. After incubation, the supernatants were collected and diluted in DMEM to determine the cytokine concentrations of interferon-gamma (IFN-γ) and interleukin 5 (IL-5) using the BD OptEIA ELISA kits (BD Biosciences, San Jose, CA, USA).

### Histopathology and determining the lung virus titer

Histopathology and virus titer in the lungs was confirmed as previously described [17, 18]. Briefly, lungs were collected at 5 days post-infection with RSV rA2 line19F. Lungs were randomly selected and immersed in 10% formalin for histopathological assay. After embedding the fixed tissues into paraffin blocks, they were sectioned for H&E or PAS staining. Stained sections were mounted onto microscope slides and visualized under the microscope. Histopathology scores were given on a scale of 0–6, based on the pathological severity. For plaque assay, lung tissue homogenates were acquired. Briefly, lung tissues were placed in a cell strainer with 1 ml serum-free DMEM media and mechanically sheared using a syringe plunger. The media portion which contains the lung cells that were filtered through the strainer was carefully collected and centrifuged at 2,000 RPM, 5 min, 4℃. After centrifugation, supernatants were collected and stored at -80℃ until use. In brief, the lung supernatants were serially diluted in serum-free DMEM media and inoculated into a confluent monolayer of HEp-2 cells cultured in 24-well plates. Plates were incubated at 37℃ for 1 h with 5% CO_2_. After aspirating the diluted mixtures, cells were overlaid with 1% noble agar and incubated for 3–4 days at 37℃ with 5% CO_2_. Agar layers were gently removed with running tap water and cells were fixed with a fixative solution containing equal volumes of acetone and methanol. After blocking with 5% skim milk prepared in PBST, cells were sequentially incubated with monoclonal anti-mouse RSV fusion protein antibody and HRP-conjugated anti-mouse IgG antibodies (1:2,000 dilution in PBS for both). The plaques were developed using 3,3’-diaminobenzidine (Invitrogen, MA, USA), and brown precipitates were counted under the microscope.

### Statistical analysis

All parameters were recorded for individuals in all the groups. The data were presented as mean ± SD, and statistical significances between groups were denoted with an asterisk. Significant differences between the means of each group were analyzed by one-way analysis of variance (ANOVA) with Tukey’s *post hoc* test and Student’s *t*-test using GraphPad Prism version 6.0 (GraphPad Software, San Diego, CA, USA). *P* values (* < 0.05, ** < 0.01) were considered statistically significant.

## Results

### Characterization of the VLPs

**Fig. 1:**
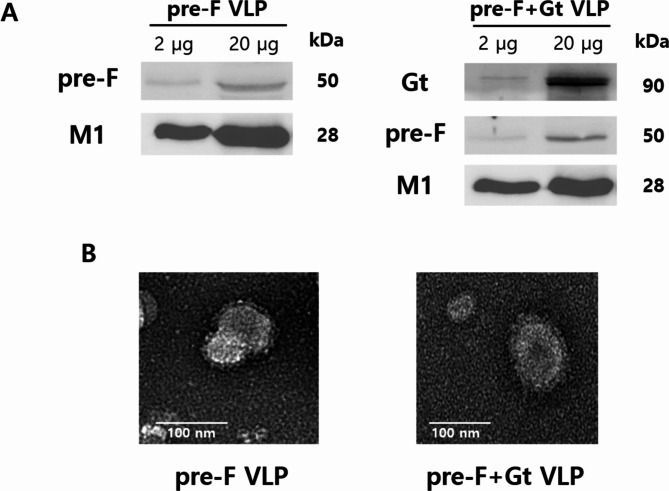
Characterization of the VLPs. The RSV pre-F and pre-F + Gt VLPs were generated using Sf9 cells using the baculovirus expression system. To confirm successful particle assembly, western blotting and TEM analyses were performed. Membranes were probed with monoclonal RSV F, monoclonal influenza M1, or RSV-positive mice sera (**A**). Transmission electron microscopy was performed to visualize VLPs (**B**)

RSV antigens displayed on the surface of the VLPs were characterized through western blot and TEM. When the membranes were probed with the monoclonal RSV F antibody, bands at 50 kDa were detected from both pre-F and pre-F + Gt VLPs. M1 expression was confirmed in both VLPs at 28 kDa. To confirm successful expression of the Gt antigen in the pre-F + Gt VLPs, membranes were probed with anti-RSV sera. Bands corresponding to the Gt antigen with the molecular weight of 90 kDa was observed in the pre-F + Gt VLPs (Fig. 1A). The morphological features of the VLPs were visualized under TEM (Fig. 1B). Pre-F and Gt antigen spikes, depicted as a dark border around the circular nanoparticles, were confirmed and suggested that the VLPs were successfully generated.

### IgG, IgA antibody response, and virus-neutralizing activity in sera

**Fig. 2:**
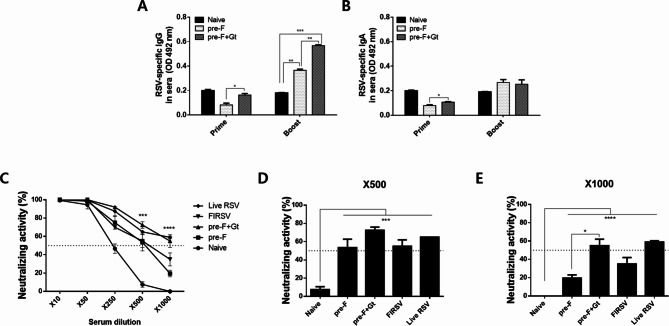
RSV-specific serum antibody responses and virus neutralization. Sera were collected from mice 4 weeks after each immunization. RSV-specific IgG (**A**) and IgA (**B**) antibody responses against FI-RSV antigens were measured by ELISA. Serum-mediated virus neutralization assay was performed using the sera acquired after the second immunization. Serially diluted sera were incubated with 50 pfu of RSV rA2-line19F virus (**C**). Virus neutralization at 1:500 (**D**) and 1:1,000 (**E**) serum dilutions were further evaluated. Data are presented as mean ± SD from experiments performed in triplicate (* *p* < 0.05, ** *p* < 0.01, *** *p* < 0.001)

Sera were collected at regular intervals post-immunization and ELISA was performed to assess virus-specific antibody responses. Priming mice with the VLPs did not elicit noticeable increases in RSV-specific IgG antibody responses in the sera. However, pre-F + Gt VLP immunization elicited significantly greater virus-specific IgG than pre-F immune sera when primed, albeit both of the VLP immune sera IgG absorbance values being below that of naïve sera. Upon boost immunization, noticeable increases in RSV-specific IgG were detected. While both VLPs elicited significantly higher RSV-specific IgG than naïve control, pre-F + Gt VLP-induced antibody responses were significantly greater than those evoked through pre-F VLPs (Fig. 2A). Similar to IgG, priming mice with the VLPs hardly elicited any changes to virus-specific IgA antibodies. Although their levels were partly enhanced after boost immunization, changes were marginal at best and significant differences compared to control were not observed (Fig. 2B). To determine virus-neutralizing antibody responses, an in vitro plaque reduction assay was performed using the sera acquired after boost immunization. As shown in Fig. 2C-E, pre-F VLPs, pre-F + Gt VLPs, FIRSV, and live RSV showed neutralizing activities at 1:500 and 1:1000 serum dilutions whereas no neutralizing activity was found from the naïve (*** *p* < 0.001, **** *p* < 0.0001). High background signals were detected from naïve sera at 1:10 and 1:50 serum dilutions. Importantly, at 1:1000 serum dilutions, pre-F + Gt vaccination showed a significantly higher titer of neutralizing activity compared to pre-F (Fig. 2E), indicating pre-F + Gt VLP immunization can induce functional antibodies to RSV.

### Antibody-secreting cell responses

**Fig. 3:**
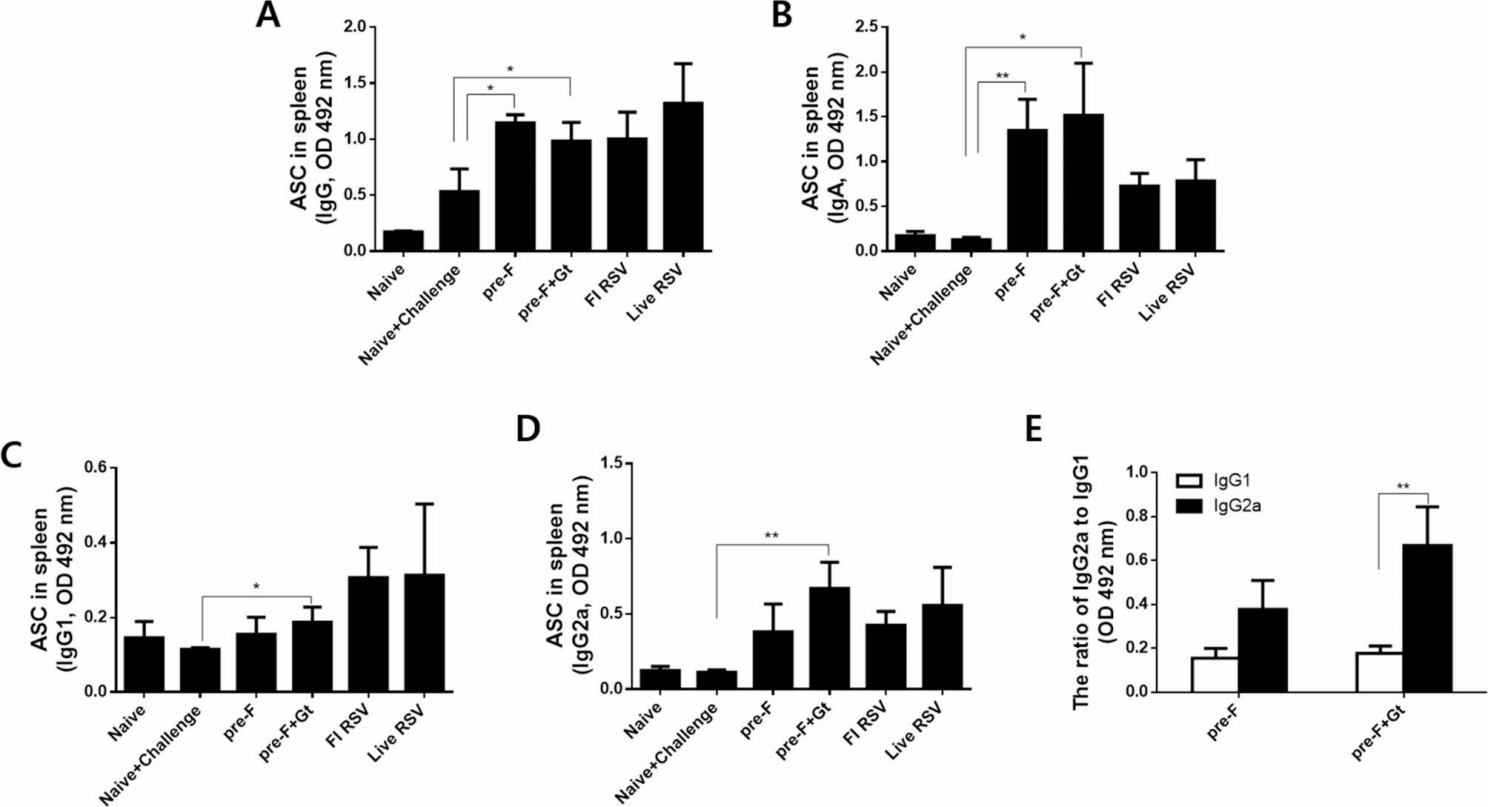
Splenic antibody-secreting cell response measurement. Splenocytes were collected at 5 days post-challenge infection and co-cultured with RSV F and G antigens. After culturing the cells for 5 days, splenic IgG (**A**), IgA (**B**), IgG1 (**C**), IgG2a (**D**), and IgG2b (**E**) antibody responses were measured via ELISA. Data are presented as mean ± SD from experiments performed in triplicate and statistical significance was determined by one-way ANOVA with Tukey’s multiple comparisons *post hoc* test (* *p* < 0.05, ** *p* < 0.01, *** *p* < 0.001)

ASC responses from splenocytes cultured with RSV F + G protein mixtures were evaluated by ELISA. As shown in Fig. 3AB, pre-F and pre-F + Gt VLPs immunizations enhanced the induction of splenic IgG and IgA ASC compared to naïve + challenge controls (*P < 0.05) (Fig. 3AB). Immunization with pre-F + Gt VLPs showed significantly higher levels of both IgG1 and IgG2a ASC responses compared to naïve + challenge control, whereas pre-F VLP-induced IgG1 and IgG2a ASC were not significantly higher (*P < 0.05, **P < 0.01) (Fig. 3CD). Importantly, pre-F + Gt VLPs induced significantly higher levels of IgG2a ASC compared to IgG1 ASC (Fig. 3E). These results indicate that pre-F + Gt VLP is highly immunogenic and induces IgG2a-dominant responses.

### Pulmonary antibody induction, T cell responses, and eosinophilia assessment

**Fig. 4:**
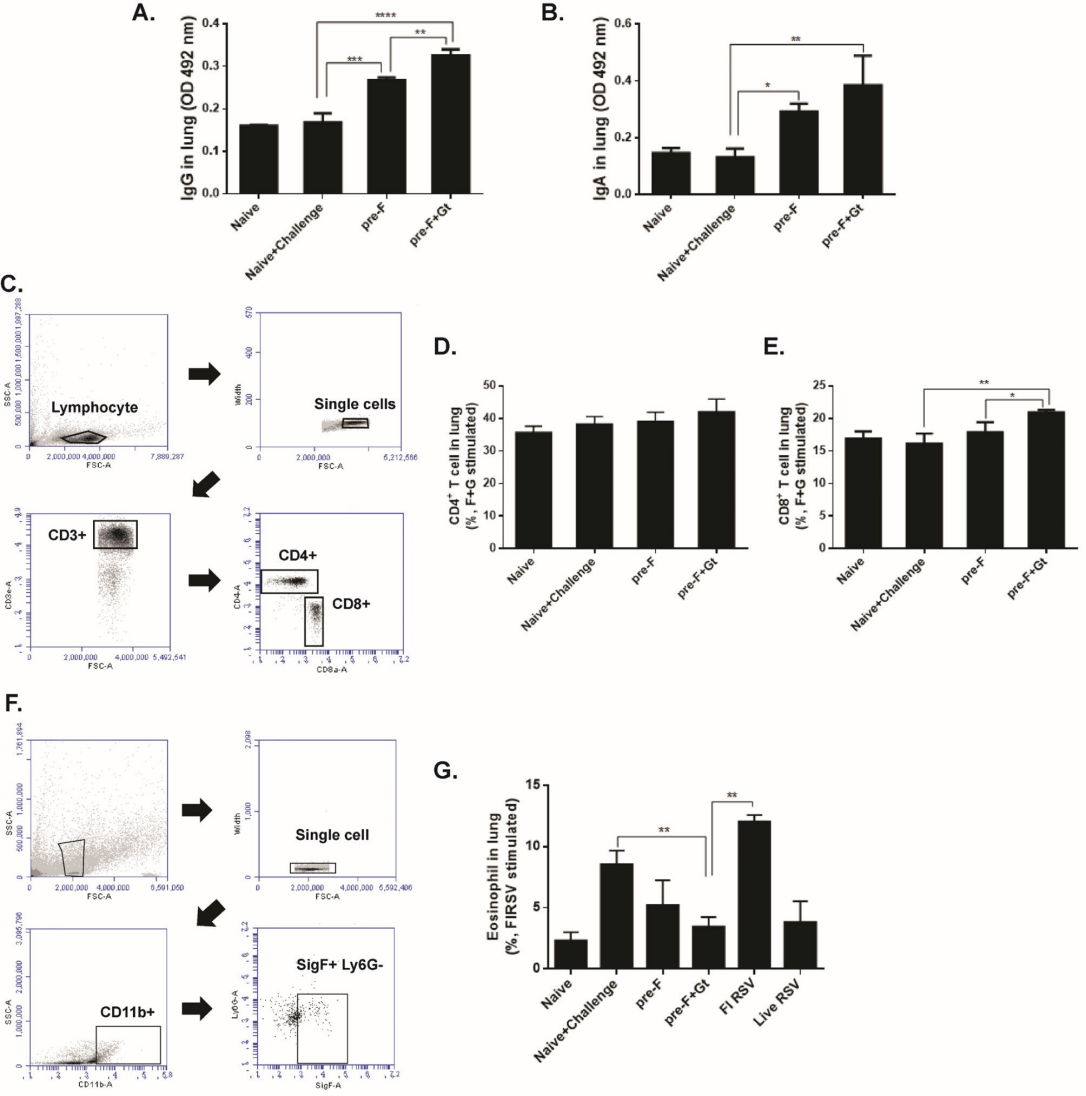
Pulmonary virus-specific antibody, T cells, and eosinophil response evaluation. To confirm the levels of IgG and IgA antibody responses, lung supernatant samples were collected at 5 days post-challenge infection. RSV specific-IgG (**A**) and IgA (**B**) antibody responses in the lungs were determined from diluted lung homogenates (1:10 dilution) by ELISA. Lung cells were isolated and stimulated with RSV antigens prior to flow cytometry analysis (**C**). Cells were gated accordingly to quantify the changes in RSV-specific CD4^+^ and CD8^+^ T cell populations (**D, E**). Eosinophil influx into the lungs was quantified by flow cytometry following RSV rA2-line19F infection. Cells were stained with CD11b, Siglec F, and Ly6G antibodies and gated (**F**). Mean values of eosinophil percentages were presented for all groups used in this study (**G**). Data are presented as mean ± SD from experiments performed in triplicate and statistical significance was determined by one-way ANOVA with Tukey’s multiple comparisons *post hoc* test (* *p* < 0.05, ** *p* < 0.01, *** *p* < 0.001, **** *p* < 0.0001)

Pulmonary antibody responses were detected from lung homogenates. RSV infection incurred negligible changes in naive controls, but substantial increases in IgG levels were observed in VLP-immunization groups (Fig. 4A). Of the two VLPs, pre-F + Gt VLPs elicited significantly greater IgG levels than pre-F VLPs. A somewhat similar finding was observed for RSV-specific pulmonary IgA responses, albeit statistical significance between the absorbance readings of the two immunizations being absent (Fig. 4B). VLP-induced CD4^+^ and CD8^+^ T cells in the lungs were assessed using flow cytometry (Fig. 4C). Despite the immunization, only marginal increases in pulmonary CD4^+^ T cell influx were observed. Specifically, neither pre-F nor pre-F + Gt VLPs elicited a significant increase in CD4^+^ T cells (Fig. 4D). Pre-F VLPs induced negligible CD8^+^ T cell influx into the lungs. Yet, pre-F + Gt VLP immunization resulted in a significant increase in pulmonary CD8^+^ T cells (Fig. 4E, * *p* < 0.05). Lung samples were carefully harvested 5 days post-challenge infection and eosinophil influx into the lungs was assessed via flow cytometry. Single cell populations of lung cells were gated using appropriate surface markers (Fig. 4F). As expected, severe eosinophilia was observed in the FI-RSV group, with a slightly lesser degree of eosinophilia in the naïve + challenge group (Fig. 4G). Compared to naïve + challenge group, reduced eosinophilia was observed in VLP immunization groups. While the means were not significantly different between naïve + challenge and pre-F, eosinophilia were significantly reduced in the pre-F + Gt VLP immunization group compared to both naïve + challenge and FI-RSV groups.

### Splenic cytokine induction in immunized mice

**Fig. 5:**
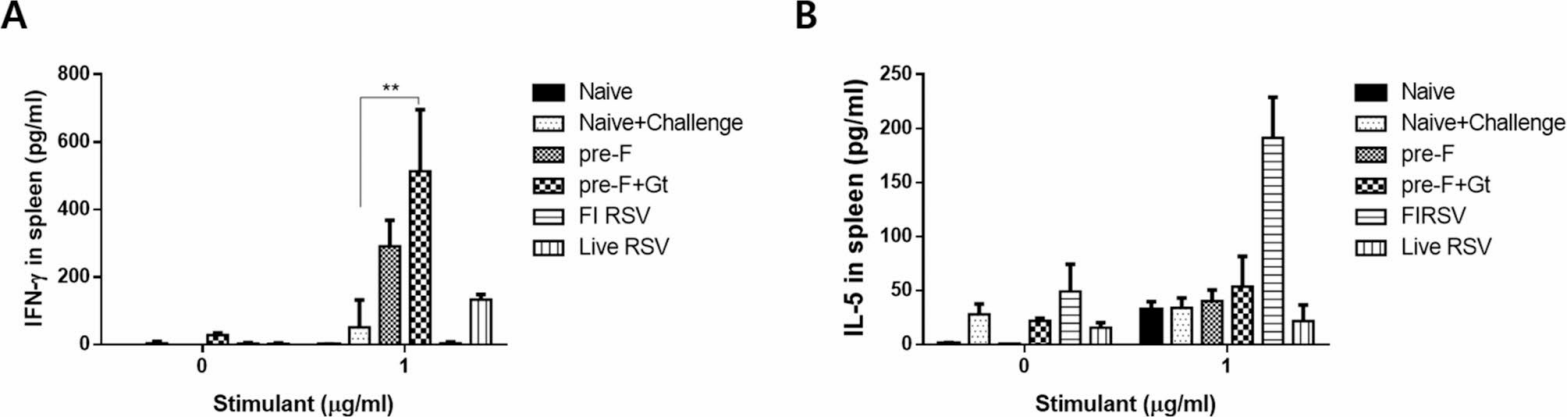
Splenic Th1 and Th2 cytokine measurement. Spleen tissues were homogenized and the splenocytes were cultured with RSV F + G protein mixture for 5 days. Representative Th1 and Th2 cytokines IFN-γ (**A**) and IL-5 (**B**) were determined using the cytokine ELISA kits. Data are presented as mean ± SD from experiments performed in triplicate and statistical significance was determined by one-way ANOVA with Tukey’s multiple comparisons *post hoc* test (* *p* < 0.05)

To assess whether VLP immunization affected the Th1/Th2 balance, splenocytes were cultured with RSV F and G protein mixtures for 5 days and supernatants were collected to determine cytokine production. When unstimulated, hardly any IFN-γ production was observed across all groups. However, stimulating the splenocytes with RSV F and G proteins resulted in a substantial increase in IFN-γ production. IFN-γ concentrations were undetectable for naïve and FI-RSV groups, while marginal production was confirmed from naïve + cha. Splenocytes of VLP-immunized mice resulted in substantially enhanced IFN-γ production compared to both naive control and live RSV-positive control groups. However, when compared to the naïve + challenge group, statistical significance was only observed in the pre-F + Gt VLP group (Fig. 5A, ** *p* < 0.01). As with IFN-γ, IL-5 was produced at negligible levels for all groups under unstimulated conditions. Even upon stimulation with the RSV F and G proteins, not many changes were induced for IL-5 production with FI-RSV being the sole exception (Fig. 5B). FI-RSV immunization incurred marked increase in IL-5 production while VLP or live RSV immunization failed to do so.

### Lung virus titer and pulmonary eosinophilia quantification

**Fig. 6:**
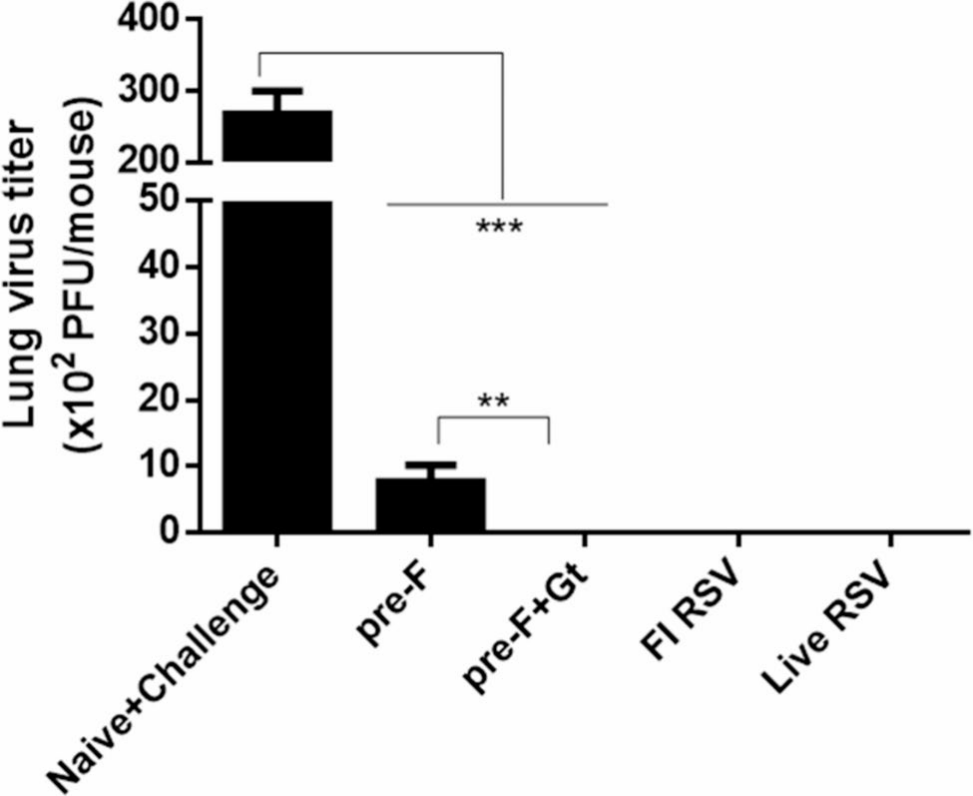
Quantifying lung virus titer reduction following immunization. Lung tissue homogenates were collected and serially diluted in serum-free DMEM prior to plaque assay. Diluted virus mixtures were inoculated into confluent monolayers of HEp-2 cells to initiate virus infection. RSV rA2-line19F plaques were visualized using the DAB substrate. Plaques were counted and used to calculate the lung virus titer from each sample. Data are presented as mean ± SD from experiments performed in triplicate and statistical significance was determined by one-way ANOVA with Tukey’s multiple comparisons *post hoc* test (** *p* < 0.01, *** *p* < 0.001)

Lung samples were carefully harvested 5 days post-challenge infection to determine pulmonary virus titer and enumerate the eosinophil influx into the lungs. RSV plaques were visualized via immunostaining. As anticipated, plaque formations were largely detected in the naïve + challenge group (Fig. 6). In contrast, plaque formations were reduced or undetectable in the lung homogenates of immunized mice. Noticeable differences were observed between the pre-F and the pre-F + Gt VLPs. Mean lung virus titers from the pre-F VLP group were approximately 1 × 10^3^ pfu, whereas virus titers were undetectable in the pre-F + Gt groups as well as in the live RSV group. A visual representation of the immunostained RSV plaques were also provided [see Additional file 1].

### Histopathology in lung

**Fig. 7:**
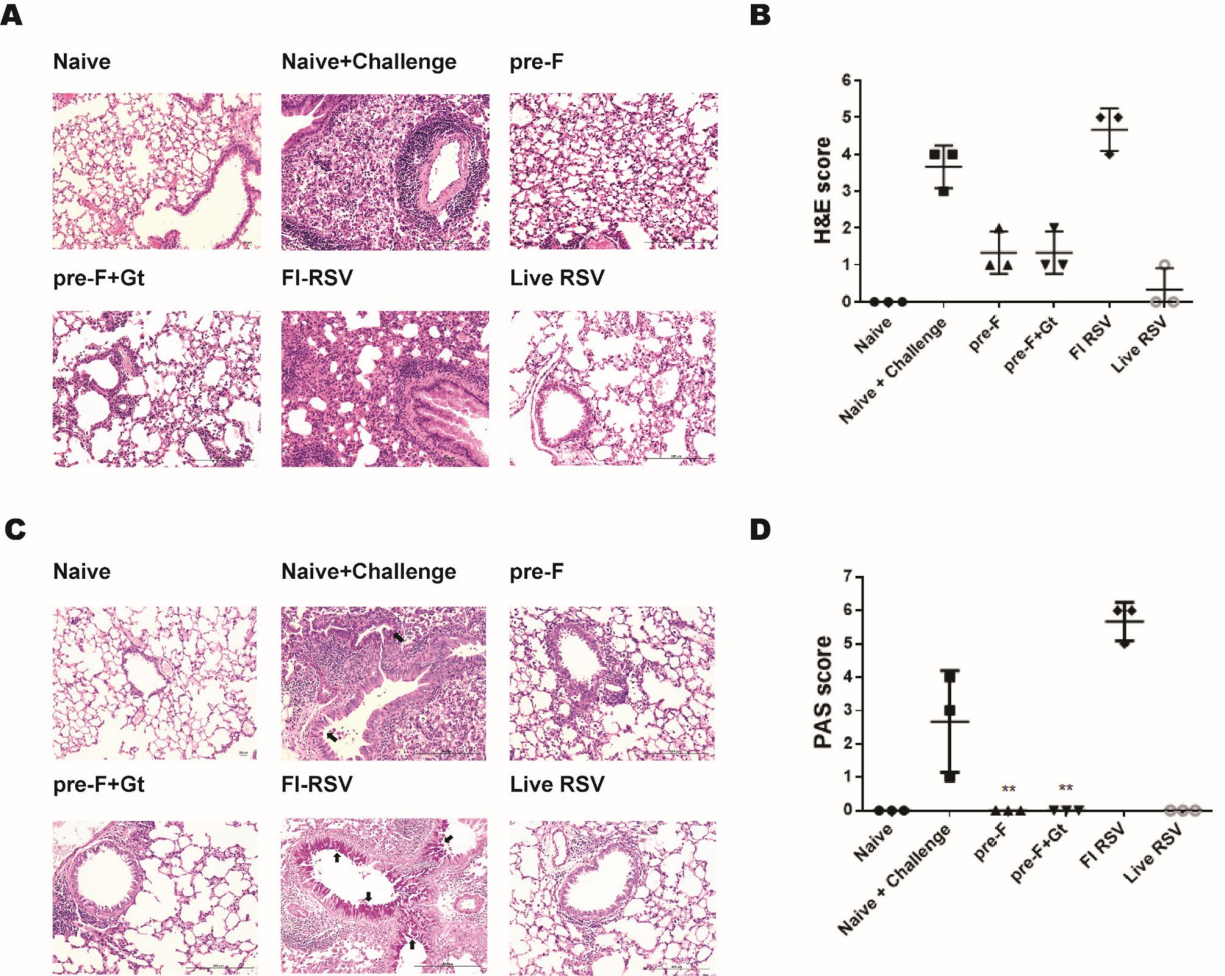
H&E and PAS staining to evaluate pulmonary histopathological changes in mice. Lung tissues were collected from RSV-infected mice and tissues were subsequently stained to visualize the pulmonary damage inflicted by RSV rA2-line19F infection. H&E stained representative lung tissue cross-sections from each group were provided (**A**) and cross-sections were scored based on the disease severity (**B**). PAS staining was also performed to confirm mucin secretion in the lungs of mice (**C**) and random fields of view were scored based on the scoring criteria (**D**). Scale bars indicate 200 μm. Black arrows pointing at the magenta-colored regions indicate mucin production. Data are presented as mean ± SD and statistical significance was determined by one-way ANOVA with Tukey’s multiple comparisons *post hoc* test (** *p* < 0.01 compared to naïve + challenge)

Stained paraffin sections were viewed under the microscope to evaluate pulmonary histopathology. Fields of views from individual cross-sections were randomly selected and blindly scored. Cellular influx was predominantly observed in naïve + challenge and FI-RSV groups (Fig. 7A). In the VLP-vaccinated mice, cellular influx occurred to a significantly lesser extent (Fig. 7B). PAS stained images depicting mucin production were performed. Similar to the H&E staining results, magenta-colored mucin production indicated using arrows was largely observed in naïve + challenge and FI-RSV groups (Fig. 7C). For the other immunization groups, histopathologies were similar to those of naive controls. In line with this trend, histopathology scores for the immunization groups were generally comparable to that of naïve control, with FI-RSV being the sole exception (Fig. 7D).

## Discussion

Tremendous global efforts to understand RSV pathogenesis contributed to much desired improvements in vaccine development, eventually paving the path to the emergence of pre-F candidate antigen. In this study, we further evaluated the efficacy of the pre-F and Gt antigen-expressing VLPs used in our previous study against a different RSV strain that better reflects clinical RSV infections. Our study demonstrated that VLPs co-expressing the pre-F and Gt antigens can provide protection against RSV rA2-line19F. Immunizing mice with these VLPs ensured the induction of RSV-specific antibody response while limiting viral replication and pulmonary histopathology.

To date, several interesting findings involving prophylaxis against RSV rA2-line19F have been reported. Treating mice with anti-RSV G protein monoclonal antibody 131-2G prior to rA2-line19F infection inhibited pulmonary inflammation, airway mucin production, and viral replication [22]. Furthermore, BALB/c mice treated with this anti-G monoclonal antibody were generally better protected against RSV rA2-line19F than those receiving the Palivizumab-like monoclonal antibody treatment [23]. Yet, to the best of our knowledge, only one study attempted to investigate the efficacy of pre-F antigens against the RSV line 19 strain. Specifically, administering pre-F protein subunit vaccines with Th2-biased adjuvants in mice exacerbated the enhanced respiratory disease development following the RSV line 19 challenge, even though protection was conferred [15]. Although the present study did not investigate various immune correlates associated with protection as described in the aforementioned study, the general consensus seems to be that pre-F antigen can confer protection against the RSV rA2-line19F strain. Current findings are also similar to those reported in our previous study [17], as pre-F + Gt VLP immunization mounted protection against rA2-line19F challenge infection and mitigated the formation of pulmonary pathologies.

The metastable pre-fusion form of the RSV F protein is crucial for neutralizing antibody response induction [24, 25]. Among them, site Ø specifically found in the pre-F protein was determined as a major target for neutralizing antibody response [26–28]. Based on this notion, we anticipated that strong neutralizing antibody responses would be induced as pre-F antigens were expressed either with or without the Gt antigens on the VLPs. Consistent with our previous study which demonstrated serum-mediated RSV A2 neutralization, RSV rA2-line19F neutralization was also observed in the present study. Discrepancies were detected when compared with another study, which recently investigated the efficacy of the adjuvanted pre-F vaccine against RSV line 19. Surprisingly, in mice receiving unadjuvanted pre-F antigen as vaccines, measurable levels of neutralizing antibody titers were only detected from half of the mice in the group [15].

Earlier studies have delineated that priming mice with FI-RSV enhances the expression of chemokines and cytokines associated with Th2 immunity, which also results in increased eosinophil influx and pulmonary inflammation [29–31]. Therefore, shifting the host’s immune response to a Th1-biased immunity could have important implications for alleviating inflammatory response. In the present study, immunizing mice with either pre-F and pre-F + Gt inhibited pulmonary inflammation to a large extent, as evidenced by the substantially lower histopathology scores, eosinophil influx, and IL-5 production compared to the FI-RSV control group. Such Th2-biased immune responses reported here are in line with earlier studies which reported aberrant immune responses that were skewed to Th2 immunity in FI-RSV mice [32, 33]. ASC response assessment revealed that the FI-RSV group produced larger quantities of Th2-associated IgG1 antibodies in mice. On the contrary, they were produced to a lesser extent in both pre-F and pre-F + Gt groups. The Th1-associated IgG2a and IgG2b were mostly produced in live RSV immunization and VLP-immunized mice. Although some IgG2a and IgG2b were detected from FI-RSV groups, changes were negligible when compared to naïve + challenge controls. Pre-F + Gt VLPs elicited greater splenic IFN-γ than those expressing the pre-F antigen alone. This was partly expected based on the previous findings reported by another research group, whose work revealed the shifting of the immune response towards Th1 immunity in mice receiving anti-RSV G antibody (131-2G) treatment prior to rA2-line19F infection. Consequently, this led to increased IFN-γ-producing T cells [34]. Consistent with this finding, splenic IFN-γ levels were markedly greater in mice immunized with the pre-F + Gt VLPs in comparison to pre-F VLPs.

It is widely accepted that CD8^+^ T cells are important regulators for limiting CD4^+^ T cell-driven immunopathology in lung airways, as described in detail by numerous earlier studies [3]. An interesting association between CD8^+^ T cells and RSV F antigen epitopes was previously discovered. The H-2K^d^-restricted epitope of RSV F protein was reported to be recognized by CD8^+^ T cells and upon primary RSV infection, the CD8^+^ effector T cells specific for this region only accounted for less than 5% of all the pulmonary CD8^+^ T cells. Contrastingly, majority of the CD8^+^ T cells induced post-infection were predominantly specific to the RSV matrix-2 protein. However, priming BALB/c mice with RSV F antigen reversed this phenomenon and elicited F protein-specific CD8^+^ T cells [35]. In a follow-up study, the same research group revealed that RSV infection selectively impairs pulmonary CD8^+^ T cell responses by interfering with T cell memory development, thus resulting in short-lived protective immunity against the virus [36]. Based on the results of our study and these earlier findings, VLP immunization in mice likely contributed to the development of robust effector CD8^+^ T cell responses that counteracted the Th2-biased inflammatory response and pulmonary virus replication. The amino acids at positions 184–198 of RSV G protein are known to predispose BALB/c mice to pulmonary eosinophilia, which seem to enhance the clonal expansion of IL-5 secreting CD4^+^ T cells [37]. Given that the Gt antigen used in this study contains this fraction, this may have possibly contributed to marginal, but not significant increase in pulmonary CD4^+^ T cell response observed in Fig. 4D.

In conclusion, we generated VLPs expressing RSV preF and preF + Gt and investigated the efficacy of protection against RSV rA2 line19F which is known to elicit more serious pulmonary inflammation. Our findings showed that pre-F + Gt VLP immunization elicited high levels of serum and lung antibody responses while maintaining the Th1/Th2 immunity balance to mitigate pulmonary inflammation, all of which contributed to less histopathological damage and lower lung virus titers following rA2-line19F challenge infection. These results further highlight the developmental potential of pre-F + Gt combinatorial antigen approach to RSV vaccines. Assessing the efficacy of this vaccine in other animal models that better reflect clinical RSV infection such as cotton rats or ferrets should be conducted to validate the in vivo findings reported here.

### Electronic supplementary material

Below is the link to the electronic supplementary material.

Supplementary Material 1

Supplementary Material 2

## Data Availability

All data generated or analyzed during this study are included in this article
