# Supplementary material for: Assessing the protection elicited by virus-like particles expressing the RSV pre-fusion F and tandem repeated G proteins against RSV rA2 line19F infection in mice
Source: Respir Res. 2024 Jan 4;25:7. doi: 10.1186/s12931-023-02641-w (PMC10765939; doi:10.1186/s12931-023-02641-w)
Supplement: Supplementary file 1 — Supplementary Material 1 [file 12931_2023_2641_MOESM1_ESM.pptx]

## Slide 1
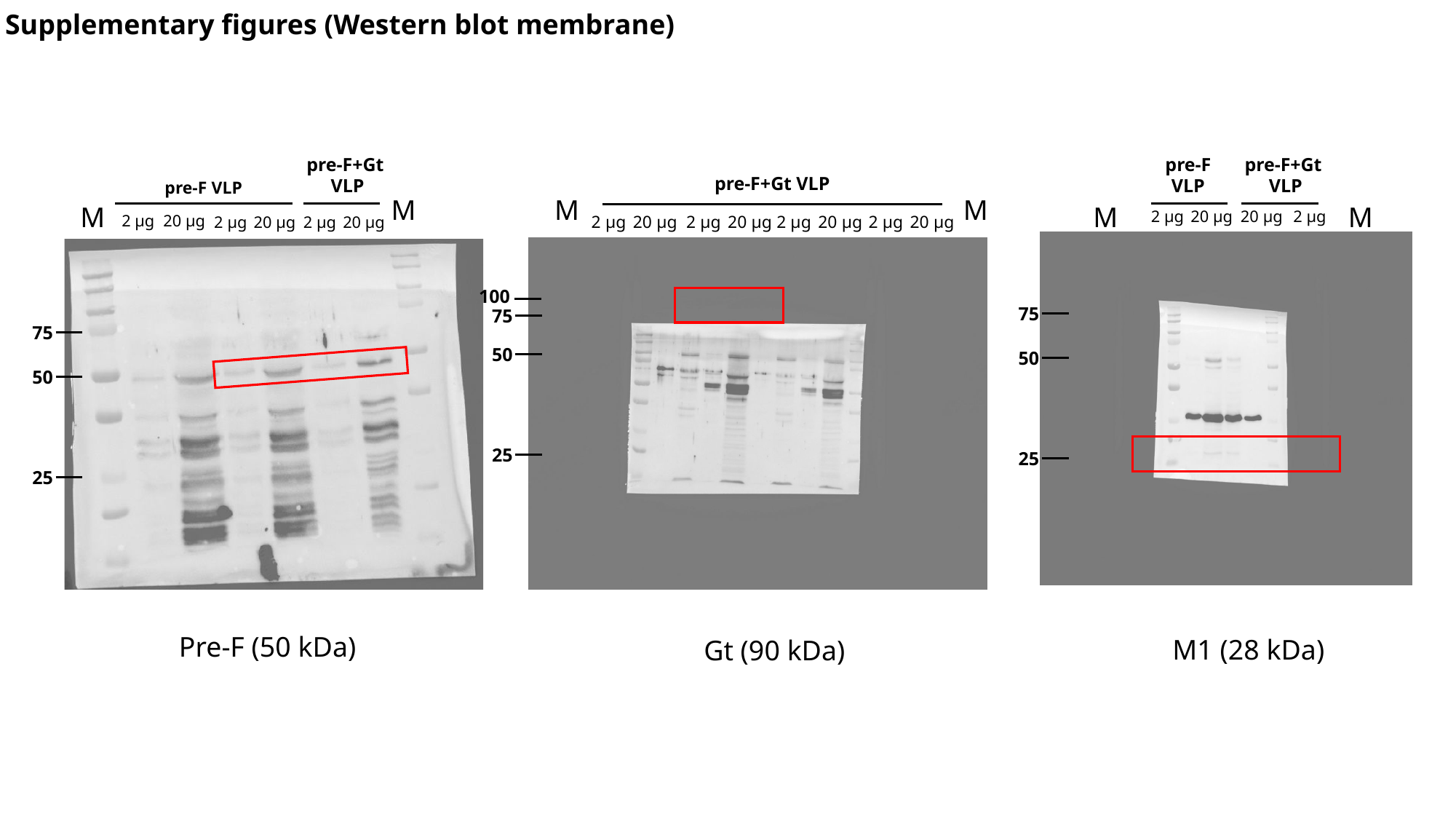

Supplementary figures (Western blot membrane)
pre-F+Gt
VLP
pre-F
VLP
pre-F+Gt
VLP
pre-F+Gt VLP
pre-F VLP
M
M
M
M
M
M
2 μg
20 μg
20 μg
2 μg
2 μg
20 μg
2 μg
20 μg
2 μg
20 μg
2 μg
20 μg
2 μg
20 μg
2 μg
20 μg
2 μg
20 μg
100
75
75
75
50
50
50
25
25
25
Pre-F (50 kDa)
M1 (28 kDa)
Gt (90 kDa)
