# Supplementary material for: Assessing the protection elicited by virus-like particles expressing the RSV pre-fusion F and tandem repeated G proteins against RSV rA2 line19F infection in mice
Source: Respir Res. 2024 Jan 4;25:7. doi: 10.1186/s12931-023-02641-w (PMC10765939; doi:10.1186/s12931-023-02641-w)

**Visual representation of the DAB-stained RSV plaques**

Images depicting RSV-infected HEp-2 cells. Brown precipitates indicate the formation of RSV plaques.


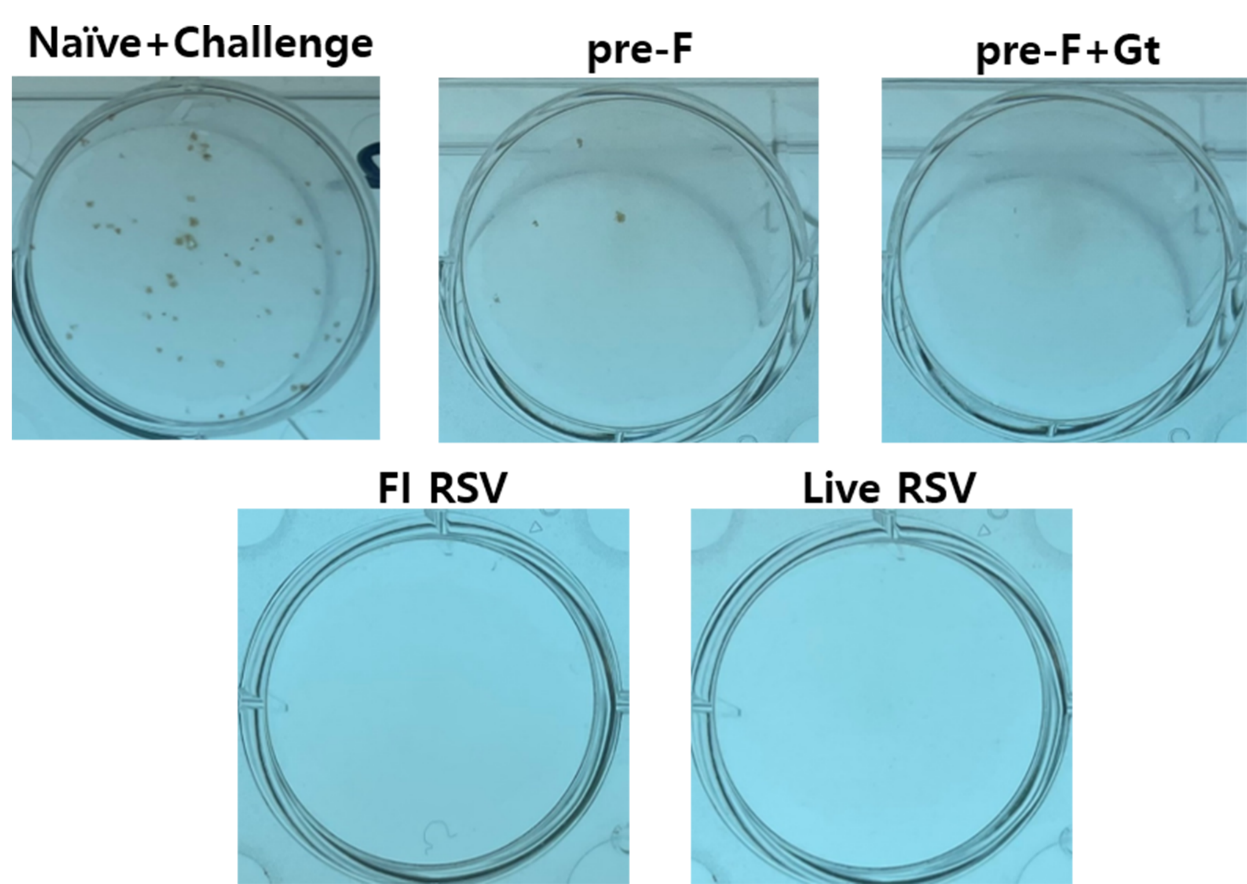

Supplement: Supplementary file 2 — Supplementary Material 2 [file 12931_2023_2641_MOESM2_ESM.docx]
